# Analysis of clinical and biochemical characteristics and left ventricular hypertrophy in patients with indeterminate saline infusion test results

**DOI:** 10.3389/fendo.2024.1506814

**Published:** 2024-12-06

**Authors:** Huiyun Qu, Jingge Zhao, Lulu Wang, Huiyu Du, Qinghui Zhang, Tingxi Sun, Chen Zhang, Jiaxuan Chen, Linya Guo, Qi Huang, Dandan Tian, Zhilan Liu, Yibin Hao, Min Liu

**Affiliations:** ^1^ Department of Hypertension, People’s Hospital of Henan University, Henan Provincial People’s Hospital, Zhengzhou, China; ^2^ Clinical Research Centre, Henan Provincial People’s Hospital, Zhengzhou, China; ^3^ Department of Hypertension, People’s Hospital of Zhengzhou University, Henan Provincial People’s Hospital, Zhengzhou, China; ^4^ Department of Hypertension, Henan Provincial People’s Hospital, Zhengzhou, China; ^5^ Department of Hypertension, Henan Provincial People’s Hospital, Henan University of Chinese Medicine, Zhengzhou, China

**Keywords:** primary aldosteronism, saline infusion test, autonomous aldosterone secretion, target organ damage, left ventricular hypertrophy

## Abstract

**Introduction:**

The clinical biochemical characteristics and target organ damage (TOD) in patients with plasma aldosterone concentrations (PAC) ranging from 50 to 100 ng/L after a saline infusion test (SIT) have not been fully studied.

**Methods:**

A total of 611 hypertensive patients with an elevated aldosterone-to-renin ratio (ARR) who underwent a supine SIT at Henan Provincial People’s Hospital were enrolled. The patients were divided into three groups according to their post-SIT PAC: <50 ng/L (control group), 50–100 ng/L (indeterminate post-SIT results group), and >100 ng/L (PA group). The clinical and biochemical characteristics and TOD of the three groups were compared. The correlation of the post-SIT PAC with left ventricular mass index (LVMI) was assessed via regression analysis.

**Results:**

The indeterminate post-SIT results group had the youngest patients and the shortest duration of hypertension. The prevalence of renal impairment (RI) and left ventricular hypertrophy (LVH) was lower than that in the PA group (P<0.05), but there was no statistically significant difference from the control group (P>0.05). After adjustment for confounders, the risk of developing carotid plaque was greater in the indeterminate post-SIT results group than in the control group (OR 1.73, 95% CI: 1.11, 2.69), and the prevalence of RI and LVH tended to increase with increasing post-SIT PAC levels. In multiple regression analyses, LVMI was significantly correlated with post-SIT PAC (P<0.05), but the basal PAC, plasma renin activity, and ARR did not significantly correlate with LVMI (P>0.05).

**Conclusion:**

A post-SIT PAC of 50–100 ng/L may be indicative of an early form of PA, and it may serve as an independent predictor of LVH, which could be related to the level of autonomously secreted aldosterone.

## Introduction

Primary aldosteronism (PA) is the most common cause of secondary hypertension, accounting for approximately 20% of refractory hypertension cases (1). It is characterized mainly by renin-independent autonomous secretion of aldosterone, leading to overactivation of the mineralocorticoid receptor. In addition to water and sodium retention, excess aldosterone has direct pro-inflammatory and profibrotic effects, resulting in target organ damage (TOD) manifesting as vascular, renal, and cardiac inflammation; fibrosis; and hypertrophy (2). The aldosterone-to-renin ratio (ARR) is the most commonly used method to screen for PA, and the diagnosis of PA relies on four diagnostic tests: the oral sodium loading test, the saline infusion test (SIT), the fludrocortisone suppression test, and the captopril provocation test. The SIT is a commonly used confirmatory test recommended by current guidelines, with a post-SIT plasma aldosterone concentrations (PAC) of less than 50 ng/L considered to indicate non-PA status and levels greater than 100 ng/L considered to indicate a high likelihood of PA, while values between 50 and 100 ng/L are considered to indicate indeterminate status (3).

Patients with indeterminate post-SIT results represent a relatively complex group. A German prospective study comprising 256 PA patients who underwent the SIT and data from 126 patients with an average follow-up of 1.2 ± 0.3 years confirmed that 42% of patients with a post-SIT PAC between 50 and 100 ng/L have unilateral disease (4). It can be seen that there are PA patients whose post-SIT PAC falls between 50 and 100 ng/L and who can achieve remission or even a cure through surgical treatment. Patients with indeterminate post-SIT results tend to present with low renin levels and high aldosterone levels, which is an important component of renin-independent aldosteronism and is also considered “mild PA” or a subclinical form of PA. However, most studies have focused on autonomous aldosterone secretion and the risk of developing hypertension and cardiovascular disease in normal subjects, with little mention of the biochemical characteristics and target organ damage in the aforementioned population.

PA is a strong factor positively associated with left ventricular hypertrophy (5). Relative to the basal PAC, the post-SIT PAC better reflects the amount of aldosterone secreted autonomously by the adrenal gland. Previous studies have examined the relationships among left ventricular LV mass index (LVMI), proteinuria (6, 7), and the post-SIT PAC in patients with PA, but these studies have often been limited to patients with PA, excluding those with indeterminate post-SIT results who were suspected of having PA. In this cross-sectional study, we analyzed the clinical biochemical characteristics and target organ damage of patients with indeterminate status (an aldosterone level of 50–100 ng/L after the supine SIT), aiming to provide a basis for the diagnosis, treatment, and prevention of hypertension and its complications in patients with indeterminate post-SIT results, and to explore the correlation between the risk of developing left ventricular hypertrophy (LVH) and post-SIT PAC.

## Methods

### Study subjects

**Figure 1:**
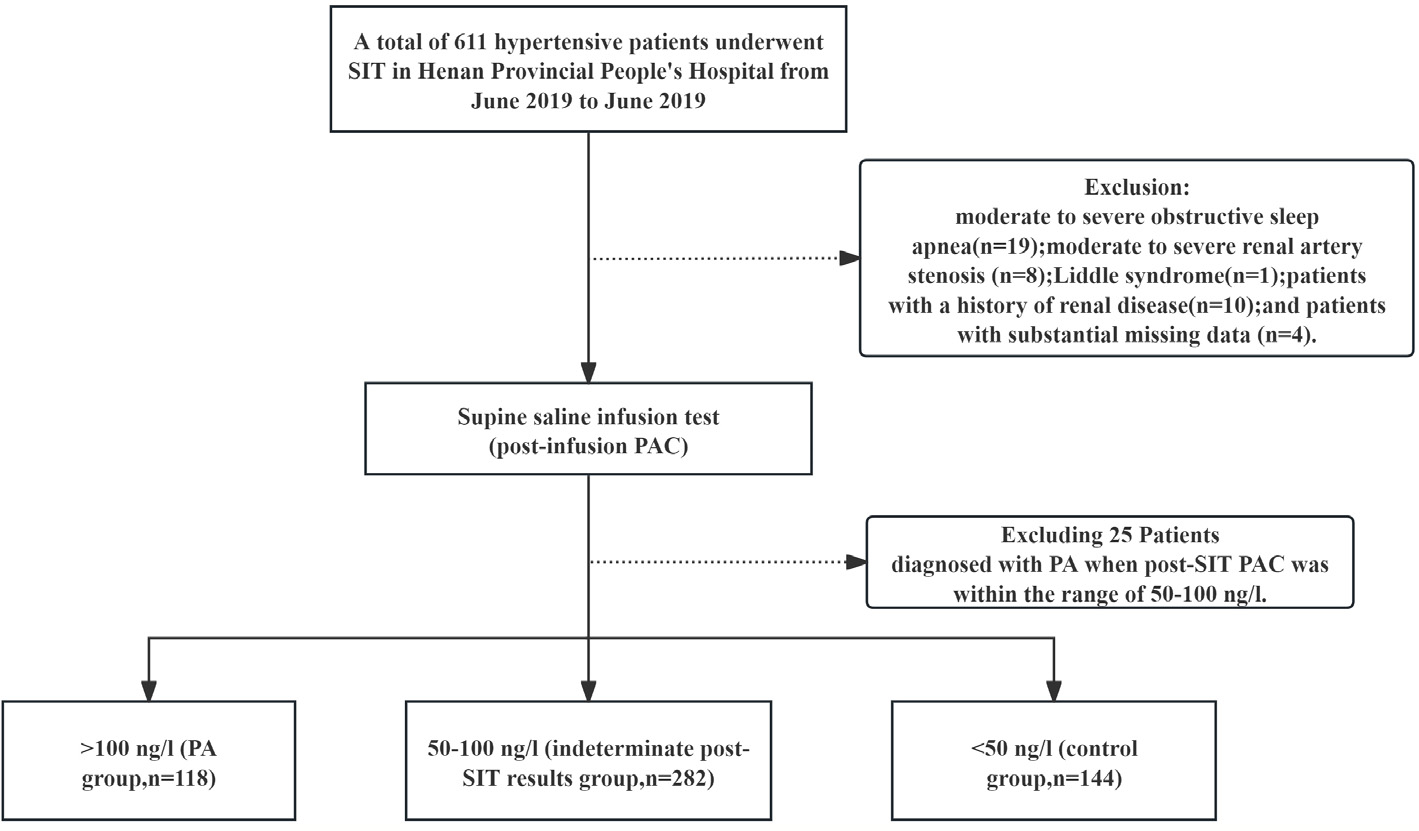
Flowchart of the study.

Among the 611 hypertensive patients with elevated ARR who underwent a SIT at Henan Provincial People’s Hospital from June 2021 to June 2023, 544 patients were screened according to the inclusion and exclusion criteria. The process of selecting the research subjects is shown in **Figure 1**. There were 144 patients with a PAC of less than 50 ng/L after the SIT (control group), 312 patients with a post-SIT PAC between 50 and 100 ng/L (indeterminate post-SIT results group), and 118 patients with a post-SIT PAC greater than 100 ng/L (PA group). The inclusion criteria were as follows: age ≥ 18 years and meeting the diagnostic criteria for hypertension according to the Chinese Guidelines for the Prevention and Control of Hypertension (2024 Revision) (8): an SBP ≥ 140 mmHg and/or a DBP ≥ 90 mmHg based on three in-office blood pressure measurements, which were not taken on the same day and without the use of antihypertensive drugs. If a patient has a previous history of hypertension and is currently on antihypertensive medication, a diagnosis of hypertension should be made even if the blood pressure is less than 140/90 mmHg. An ARR of ≥30 (ng/dL)/[μg/(L·h)] above the screening test cutoff point was also used.

The following patients were excluded: those with secondary hypertension other than PA, such as Cushing’s syndrome and pheochromocytoma; those with a history of renal disease, such as nephritis, nephrotic syndrome and nephrectomy; those with severe organic heart disease, such as Hypertrophic cardiomyopathy, dilated cardiomyopathy; New York Heart Association class III or higher cardiac insufficiency; those who were pregnant or lactating; those who were using hormones or oral contraceptives; those with a positive PA screening but refused to complete confirmatory testing; those who were unable to cooperate due to mental illness; those who underwent confirmatory tests other than the SIT, such as the captopril test and the fludrocortisone test; those diagnosed with PA when post-SIT PAC was within the range of 50-100 ng/l (**Supplementary Table S1**); and those with substantial missing data.

The patients with negative or indeterminate SIT results were treated according to essential hypertension. Of the 25 excluded patients with indeterminate SIT results but empirically diagnosed with PA, 9 patients agreed to undergo renal venous sampling (AVS) according to the patients’ wishes, and 2 of them underwent unilateral adrenal adenoma resection. The pathological results showed that both were adrenal cortical adenomas, and no medication was taken after surgery. The remaining 23 patients received mineralocorticoid receptor antagonists (MRA) treatment according to the treatment of PA.

### Data collection

The information collected included age, sex, body mass index (BMI), duration of hypertension, type of antihypertensive medication, history of hypokalemia, history of smoking, history of diabetes mellitus, biochemical results, hormone levels at the time of the screening test and confirmatory test, imaging data, and hypertensive target organ damage.

### Measurement and definition

All patients were required to discontinue medications that had an effect on the ARR, including b-blockers, angiotensin-converting enzyme inhibitors, and angiotensin receptor blockers, for ≥2 weeks. Spironolactone and other diuretics were discontinued for ≥4 weeks. α1 receptor blockers and nondihydropyridine calcium channel blockers (verapamil or diltiazem) were permitted for blood pressure control. In patients with hypokalemia, potassium was replaced with oral potassium supplementation to normalize blood potassium levels prior to screening. Blood samples were collected according to the 2016 American Endocrine Society guidelines for the diagnosis and treatment of primary aldosteronism: blood was collected after the patient had been up (sitting, standing, or walking) for at least 2 hours and had rested for 5 to 15 minutes; blood was delivered under conditions that maintained room temperature to prevent the conversion of inactive renin to active renin; and plasma was frozen and stored immediately after centrifugation (3).

### Saline infusion test

Beginning at 8:00 a.m., patients were instructed to rest in bed for more than 1 hour, during which 2000 ml of saline was infused over 4 hours. Blood was collected to detect PAC and renin activity before and after saline infusion, respectively. Throughout the entire process, the patients remained fasting and were kept in a recumbent position, and they were monitored for vital signs such as blood pressure and heart rate. The plasma aldosterone concentration and plasma renin activity were determined by radioimmunoassay (counter model MGLM20) in the laboratory of nuclear medicine, using the instrument and plasma aldosterone concentration kit from Shenzhen New Industry Biomedical Engineering Cooperation.

The diagnosis of PA was based on a positive SIT result (a post-infusion PAC >100 ng/L) or, in the case of an indeterminate SIT result (a post-infusion PAC between ≥50 and ≤100 ng/L), a diagnosis made through discussion by three professors with more than 20 years of experience in the diagnosis and treatment of PA at our center. They based their discussion on the patient’s SIT result, age, blood pressure, blood potassium level, imaging findings (i.e., the presence or absence of an adrenal adenoma), and antihypertensive medication use. These variables were repeatedly used as predictors in PA’s predictive model (9–11). We did not use other confirmation tests.

Target organ damage was defined as the presence of lesions in at least one of the following target organs: renal impairment (RI) was defined as a UACR ≥ 30 mg/g or an eGFR < 60 mL/min/1.73 m², and the estimated glomerular filtration rate (eGFR) was calculated using the CKD-EPI equation. Left ventricular hypertrophy (LVH) was defined as LVMI ≥ 115 g/m² in men and LVMI ≥ 95 g/m² in women (12). Carotid plaque was defined as the formation of carotid atheromatous plaque observed on carotid ultrasound (with limited cIMT ≥ 1.5 mm or localized increase in cIMT > 0.5 mm or 50%) (13).

### Statistical analysis

The data were analyzed using the statistical software SPSS 25.0. Measurements conforming to a normal distribution are expressed as means ± standard deviations (x ± s), and comparisons between groups were performed using one-way ANOVA with *post hoc* tests. Data that did not conform to a normal distribution are expressed as medians (interquartile spacing) [M (Q1, Q3)], and comparisons between groups were made using the Kruskal–Wallis test. Count data are expressed as frequencies and percentages, and comparisons between groups were made using the chi-square test or Fisher’s exact test (where necessary). Logistic regression was used to adjust for confounders, with sex, age, duration of hypertension, and history of diabetes mellitus included as confounders in the model and tested for trends, with the risk of an event expressed as a risk ratio (OR) with a confidence interval of 95% (95% CI). The relationship between continuous variables was assessed by calculating Spearman’s rank correlation coefficient. Statistically significant and/or clinically important variables from univariate analysis were included in the multivariable model, considering also the number of outcomes, to avoid overfitted models. Multiple linear regression analysis was performed using backward stepwise regression. A *P* value < 0.05 was considered to indicate statistical significance.

## Results

### Comparison of the basic data of the three groups of patients with a post-SIT PAC < 50 ng/L (control group), 50–100 ng/L (indeterminate post-SIT results group), or > 100 ng/L (PA group)

A total of 144 patients (26.47% of the total sample size) were in the control group, 282 patients (51.84% of the total sample size) were in the indeterminate post-SIT results group, and 118 patients (21.69% of the total sample size) were in the PA group. The clinical characteristics of the three groups of patients are shown in **Table 1**. Compared with the control group, the indeterminate post-SIT results group had a greater proportion of male patients, a younger age, a shorter duration of hypertension, lower serum potassium levels, a greater incidence of dyslipidemia, and higher diastolic blood pressure (BP), serum creatinine levels, and homocysteine levels (P < 0.05). Compared with the PA group, the indeterminate post-SIT group had a greater proportion of female patients, a shorter duration of hypertension, lower systolic BP, urinary microalbumin/creatinine ratio (UACR), and prevalence of hypokalemia; fewer types of antihypertensive medications; and a greater level of serum potassium (P < 0.05). There were no significant differences among the three groups in BMI, estimated glomerular filtration rate (eGFR), history of diabetes mellitus, cholesterol (CHOL), triglycerides (TG), low-density lipoprotein cholesterol (LDL-C), or uric acid levels (P > 0.05).

**Table 1:** Comparison of basic data among patients with different post-SIT PAC levels.

Parameter	control group(n=144)	indeterminate post-SIT results group (n=282)	PA group(n=118)	P- value
Sex(female/male)	106/38	150/132^a^	45/73^ab^	<0.001
Age(years)	52.20 ± 11.56	48.95 ± 10.92^a^	49.02 ± 10.04^a^	0.004
BMI(kg/m2)	25.39(23.31,27.48)	25.91(23.99,28.29)	26.02(24.03,28.04)	0.162
Duration of hypertension(years)	3.00(1.00,7.75)	2.00(0.23,6.00)^a^	3.00(1.00,10.00)^b^	0.002
Hypokalemia(%)	14(9.7)	41(14.5)	60(50.8)^ab^	<0.001
Ever smoker(%)	16(11.1)	48(17.0)	29(24.6)^a^	0.017
Diabetes mellitus(%)	15(10.4)	35(12.4)	18(15.3)	0.499
Systolic BP(mmHg)	142.77 ± 18.59	145.45 ± 17.02	151.08 ± 20.80^ab^	0.001
Diastolic BP(mmHg)	86.60 ± 13.82	90.45 ± 15.00^a^	93.18 ± 14.99^a^	0.001
Hypertension stage 3(%)	10(6.9)	26(9.2)	24(20.3)^ab^	0.001
Antihypertensive drug classes	1(0,2)	1(0,2)	2(1,3)^ab^	<0.001
Serum potassium(mmol/L)	4.01 ± 0.32	3.91 ± 0.34^a^	3.55 ± 0.49^ab^	<0.001
Serum sodium,(mmol/L)	141(140,142)	141(140,143)	142(141,144)^a^	0.041
CHOL(mmol/L)	4.48 ± 0.84	4.57 ± 0.96	4.38 ± 0.97	0.167
TG(mmol/L)	1.39(1.04,1.89)	1.45(1.07,2.03)	1.64(1.12,2.43)	0.053
HDL-C(mmol/L)	1.21(1.05,1.38)	1.14(0.99,1.33)^a^	1.05(0.93,1.22)^ab^	<0.001
LDL-C(mmol/L)	2.57 ± 0.65	2.67 ± 0.74	2.54 ± 0.78	0.383
Dyslipidemia(%)	55.0(38.2)	142.0(50.4)^a^	58.0(49.2)	0.036
eGFR( mL/min/1.73m2)	106.28(100.57,114.28)	106.52(97.75,114.79)	107.45(99.32,115.48)	0.984
Serum creatinine (μmol/L)	55(50,65)	64(55,74)^a^	65(57,78)^a^	<0.001
FPG(mmol/L)	5.09(4.46,5.56)	5.13(4.52,5.73)	5.16(4.61,5.84)	0.177
Glycosylated hemoglobin	5.50(5.30,5.85)	5.60(5.30,6.00)	5.60(5.30,6.10)	0.456
UACR(mg/g)	6.00(4.03,11.12)	7.08(3.48,12.18)	17.97(5.97,54.19)^ab^	<0.001
Uric acid(μmol/L)	307.0(257.0,370.5)	329.5(267.0,391.5)	334.0(261.0,405.0)	0.154
Homocysteine(μmol/L)	9.90(8.58,12.43)	11.40(9.50,13.43)^a^	11.30(9.60,13.80)^a^	0.001
PRA (ng/mL)/h)	0.2(0.2,0.3)	0.3(0.2,0.3)^a^	0.3(0.2,0.3)	0.039
PAC (pg/mL)	134.10(118.10,160.10)	157.90(131.40,188.15)^a^	235.95(182.75,315.90)^ab^	<0.001
ARR(ng/dl)/(ng/ml per h)	54.15(40.58,66.25)	55.35(41.88,73.50)	87.25(60.41,123.73)^ab^	<0.001
Pre-SIT PAR (ng/mL)/h)	0.2(0.2,0.2)	0.2(0.2,0.2)	0.2(0.2,0.3)	0.166
Pre-SIT PAC (pg/mL)	75.60(65.60,88.05)	102.90(88.68,121.53)^a^	186.60(148.95,249.98)^ab^	<0.001
Post-SIT PAR (ng/mL)/h)	0.2(0.2,0.2)	0.2(0.2,0.2)	0.2(0.2,0.2)^ab^	0.010
Post-SIT PAC (pg/mL)	42.50(38.73,46.08)	64.45(58.30,75.00)^a^	132.10(113.20,181.88)^ab^	<0.001

a: *P* < 0.05 vs. control group; b: *P* < 0.05 vs. indeterminate post-SIT results group; BMI: body mass index; CHOL, total cholesterol; TG, triglycerides; HDL-C, high-density lipoprotein cholesterol; LDL-C, low-density lipoprotein cholesterol; eGFR, estimated glomerular filtration rate; FPG, fasting blood glucose; UACR, urinary microalbumin/creatinine; PRA, plasma renin activity; PAC, plasma aldosterone concentration; ARR, aldosterone-renin ratio; pre-SIT, pre-saline infusion test; post-SIT, post-saline infusion test.

The patients in the indeterminate post-SIT results group had higher concentrations of aldosterone before and after the SIT, as well as standing plasma renin activity (PRA) and standing PAC, than those in the control group (P < 0.05). Compared with the PA group, the indeterminate post-SIT results group had significantly lower PAC before and after the SIT, as well as standing PAC and standing ARR (P < 0.001).

### Comparison of cardiac ultrasound indicators in the three groups of patients with a post-SIT PAC < 50 ng/L (control group), 50–100 ng/L (indeterminate post-SIT results group), and > 100 ng/L (PA group)

Compared with the PA group, the left ventricular posterior wall thickness (LVPWT), left ventricular end-systolic diameter (LVDs), left ventricular end-diastolic diameter (LVEDD), and LVMI in the indeterminate post-SIT results group were lower (P < 0.05). However, no significant difference was observed between the indeterminate post-SIT results group and the control group in cardiac ultrasound indicators (P > 0.05) (**Table 2**).

**Table 2:** Comparison of cardiac ultrasound indicators of patients with different post-SIT PAC levels.

Parameter	control group(n=144)	indeterminate post-SIT results group (n=282)	PA group(n=118)	P- value
IVST (cm)	0.93 ± 0.10	0.96 ± 0.14	1.00 ± 0.17^a^	0.001
LVPWT (cm)	0.91 ± 0.08	0.93 ± 0.11	0.97 ± 0.12^ab^	<0.001
LVDs (cm)	2.95 ± 0.31	2.92 ± 0.36	3.04 ± 0.38^b^	0.019
LVEDD(cm)	4.60(4.40,4.80)	4.60(4.40,4.90)	4.70(4.50,4.90)^b^	0.031
LVMI (g/m^2^)	79.52(73.98,91.39)	79.35(72.60,89.24)	88.71(75.72,100.74)^ab^	<0.001
LVEF(%)	65(62,68)	65(63,68)	65(62,68)	0.328

a: P < 0.05 vs. control group; b: P < 0.05 vs. indeterminate post-SIT results group; IVST, interventricular septum thickness; LVPWT, left ventricular posterior wall thickness; LVDs, left ventricular end-systolic diameter; LVEDD, left ventricular end-diastolic diameter; LVMI, left ventricular mass index; LVEF, left ventricular ejection fraction.

### Comparison of target organ damage in the three groups of patients with a post-SIT PAC < 50 ng/L (control group), 50–100 ng/L (indeterminate post-SIT results group), and > 100 ng/L (PA group)

Among all events, carotid plaque was the most common type of target organ damage in patients with hypertension, accounting for 41.7%, 49.3%, and 52.5% of the sample sizes in the three groups (**Table 3**). The prevalence of RI and LVH was lower in the indeterminate post-SIT results group than in the PA group (5.7% vs. 27.1%, P < 0.001; 8.9% vs. 23.7%, P < 0.001), but the prevalence of damage to each of these target organs did not differ significantly from that in the control group (P > 0.05).

**Table 3:** Comparison of target organ damage in patients with different post-SIT PAC levels.

Parameter	control group(n=144)	indeterminate post-SIT results group (n=282)	PA group(n=118)	P1 Value	P2 Value	P3 Value
left ventricular hypertrophy, n(%)	18(12.5)	25(8.9)	28(23.7)	0.239	0.017	<0.001
renal impairment, n(%)	5(3.5)	16(5.7)	32(27.1)	0.321	<0.001	<0.001
carotid plaque, n(%)	60(41.7)	139(49.3)	62(52.5)	0.136	0.079	0.553

P1: control group vs. indeterminate post-SIT results group; P2: control group vs. PA group; P3: indeterminate post-SIT results group vs. PA group.

### Comparison of the risk of target organ damage after adjusting for confounders in the three groups of patients with a post-SIT PAC < 50 ng/L (control group), 50–100 ng/L (indeterminate post-SIT results group), and > 100 ng/L (PA group)

**Table 4** shows that, after adjusting for confounders such as sex, age, duration of hypertension, and history of diabetes mellitus, the risk of carotid plaque was greater in patients in the indeterminate post-SIT results group than in the control group (OR 1.73, 95% CI: 1.11, 2.69). In the trend test, the prevalence of RI and LVH tended to increase with increasing post-SIT PAC (P < 0.001 for trend).

**Table 4:** Odd ratio after adjustment for confounding factors (sex, age, duration of hypertension and history of diabetes mellitus).

	Grouping	N(%)	OR(95% CI)	P- value	P for trend
	control group	60(41.7)	1(Reference)	⎯⎯	⎯⎯
Carotid plaque	indeterminate post-SIT results group	139(49.3)	1.73 (1.11, 2.69)	0.015	
	PA group	62(52.5)	1.86 (1.08,3.18)	0.024	
	control group	5(3.5)	1(Reference)	⎯⎯	<0.001
Renal impairment	indeterminate post-SIT results group	16(5.7)	1.35 (0.47,3.88)	0.583	
	PA group	32(27.1)	8.15(2.96,22.41)	<0.001	
Left ventricular hypertrophy	control group	18(12.5)	1(Reference)	⎯⎯	<0.001
	indeterminate post-SIT results group	25(8.9)	0.81 (0.42,1.57)	0.536	
	PA group	28(23.7)	2.69 (1.34,5.39)	0.005	

OR, odds ratio; CI, confidence interval.

### Correlation of LVMI with RAAS system-related hormones

We assessed the correlation between LVMI and RAAS system-related hormones using Spearman’s rank correlation coefficient and found that post-SIT PAC was significantly correlated with LVMI. No correlation was observed between PAC, PRA, or ARR and LVMI (**Table 5**).

**Table 5:** Univariate correlation analysis with left ventricular mass index as the dependent variable.

Parameter	*r*	*P*
PRA	0.024	0.608
PAC	0.103	0.064
ARR	0.046	0.324
Post-SIT PAC	0.124	0.007

Correlations are expressed by the Spearman coefficient. PRA, renin activity; PAC, plasma aldosterone concentration; ARR, aldosterone-renin ratio; post-SIT, post-saline infusion test.

### Correlation between LVMI and post-SIT PAC after adjustment for confounders

The association between post-SIT PAC and LVMI, a correlate of LVH, was assessed via multiple linear regression analysis. We found that post-SIT PAC was still significantly correlated with LVMI after adjusting for confounders such as sex, duration of hypertension, antihypertensive drug classes, ever smoking, systolic BP, fasting plasma glucose (FPG), Serum potassium, and hypokalemia (P < 0.05) (**Table 6**).

**Table 6:** Multivariate linear regression analysis.

	Unstandardized Coefficients		Standardized β	95% CI		*P*
	β	Standard error		Lower	Upper	
Sex	-5.500	1.674	-0.149	-8.790	-2.209	0.001
Antihypertensive drug classes	1.424	0.808	0.081	-0.164	3.013	0.079
Serum potassium	-3.839	2.180	-0.086	-8.123	0.444	0.079
FPG	1.813	0.603	0.136	0.628	2.998	0.003
Post-SIT PAC	0.024	0.010	0.119	0.005	0.043	0.014

FPG, fasting blood glucose; CI, confidence interval.

## Discussion

PA was once considered a rare disease. Saline infusion test, the most commonly used confirmatory test for PA, has a significant limitation, namely, that the optimum critical value is not clear. In recent years, studies have suggested that there is a continuum of renin-independent aldosteronism and mineralocorticoid receptor activity, ranging from mild to markedly dysregulated and autonomous, in the context of normotension (14). Markou et al. (15) proposed that 13% of the normotensive population had PA and showed that these individuals had a more than 15-fold higher risk of developing essential hypertension at 5-year follow-up. The prevalence of PA is also not uncommon in patients with relatively mild hypertension. Monticone et al. (16) found that the prevalence of PA in patients with stage 1 and stage 2 hypertension could reach 3.9% and 9.7%, respectively. This also means that what we currently recognize as severe hypertension and hypokalemic PA may be only the “tip of the iceberg” in the spectrum of renin-independent aldosteronism and excessive MR activation. Patients with renin-independent aldosteronism who present with normotension and mild to moderate hypertension are described as subclinical PA or mild PA and may progress to overt PA in the future. A prospective study enrolling 184 patients with elevated ARR and negative confirmatory tests from two hypertension centers in Torino and Munich was followed up for 2-5 years, with 20% of patients with negative confirmatory tests developing significant PA (17). In these individuals with low renin levels, autonomous secretion of aldosterone increases their risk of hypertension and cardiovascular and renal disease (15, 18, 19), and these risks can be mitigated by targeted therapies such as mineralocorticoid receptor antagonists. This study is the first to analyze target organ damage in patients with indeterminate post-SIT results.

Patients with indeterminate post-SIT results accounted for 51.84% of the enrolled population as part of renin-independent aldosteronism. Compared with those in the control and PA groups, the patients in the indeterminate post-SIT results group were the youngest and had the shortest duration of hypertension. Furthermore, 14.5% of them had a history of hypokalemia, and their serum potassium and creatinine levels were between those of the other two groups, which is not in complete agreement with the study of Cornu et al. (20). In their study, there were no significant differences in age or the duration of hypertension among the three groups of patients, which may be due to the different populations included. Additionally, the PA group was male-dominated in our study, which differs from previous studies. We consider that the screening and diagnosis of PA in women is more likely to be delayed than in men due to the influence of the menstrual cycle and hormonal contraception (21). In terms of hormones, as we predicted, the standing PAC and the pre-SIT and post-SIT PAC in patients with indeterminate post-SIT results ranged between those of the other two groups (the control and PA groups).

In a previous study, Nanfang Li et al. (22) included 1,267 hypertensive patients who underwent the SIT to explore the distribution and clinical manifestations of the main types of low-renin hypertension (LRH). They reported that primary aldosteronism, including both overt and mild PA, was the main form of LRH and that mild PA had milder clinical manifestations than overt PA. Similarly, in our study, the prevalence of RI and LVH was significantly greater in the PA group than in the other two groups. The target organ damage in the patients in the indeterminate post-SIT results group was less severe than that in the patients in the PA group and was not significantly different from that of those in the control group. However, after adjusting for common confounders such as sex, age, duration of hypertension, and history of diabetes, the risk of carotid plaque in the indeterminate post-SIT results group was 1.73 times greater than that in the control group. Additionally, trend tests revealed that the risk of RI and LVH increased with increasing post-SIT PAC levels. It follows that different levels of autonomous aldosterone secretion clearly differ in the degree of target organ damage in patients with renin-independent aldosteronism. Recently, a Japanese study evaluated the clinical characteristics of the borderline group (100≤ARR < 200 after CCT) after a captopril-challenge test and confirmed that the prevalence of target organ damage increases linearly as the aldosterone-to-renin ratio rises following the captopril challenge test (23). This reminds us again that early detection and the application of MR receptor antagonists may increase the chances of mitigating MR-mediated cardiovascular disease at an early stage.

The post-SIT PAC is strongly associated with LVH in patients with PA. In a previous study, Ohno et al. (24) demonstrated a significant correlation between plasma aldosterone concentration and LVMI after the captopril provocation test or SIT using logistic regression in patients with PA. Additionally, there was no significant correlation between basal plasma aldosterone concentration, plasma renin activity, or ARR and LVMI. This finding was also confirmed in our study, which further demonstrated that the correlation between post-SIT PAC and LVMI was not limited to patients with overt PA but was present in the entire population of patients with low-renin hypertension.

The strengths of this study include our reporting of the proportion of patients with indeterminate post-SIT results and our analysis of the clinical and biochemical characteristics of this population, as well as the target organ damage, for the first time. Additionally, we analyzed the trend in the risk of target organ damage in patients with indeterminate post-SIT results after adjusting for confounders. This study has several limitations. First, this was a single-center retrospective study, and although the disease characteristics of patients with indeterminate post-SIT results were preliminarily explored, further investigation into the factors influencing the outcomes of these patients is needed. Second, the confirmatory test performed in our center was a supine SIT instead of a seated, but this does not negate the associations shown in our study. Furthermore, the population enrolled in this study underwent a single confirmatory trial, and late follow-up is currently underway.

## Data Availability

The data analyzed in this study is subject to the following licenses/restrictions: The data during the current study are not publicly available due to privacy protection issues but are available from the corresponding author on reasonable request. Requests to access these datasets should be directed to ML, liumin136@126.com.
