# Supplementary material for: Analysis of clinical and biochemical characteristics and left ventricular hypertrophy in patients with indeterminate saline infusion test results
Source: Front Endocrinol (Lausanne). 2024 Dec 6;15:1506814. doi: 10.3389/fendo.2024.1506814 (PMC11658999; doi:10.3389/fendo.2024.1506814)
Supplement: Supplementary file 1 [file Table1.docx]

| Serial number | Sex | Age | Hypokalemia | Adrenal adenoma | Taking 3 or more antihypertensive drugs | Complication | AVS | AVS results | Pathological results |
| --- | --- | --- | --- | --- | --- | --- | --- | --- | --- |
| 1 | Male | 63 | √ | √ | √ | √ | √ | Lateralized |  |
| 2 | Female | 63 |  | √ | √ | √ |  |  |  |
| 3 | Female | 41 | √ | √ | √ | √ | √ | Nonlateralized |  |
| 4 | Male | 57 |  | √ | √ | √ | √ | Lateralized | Adrenal cortical adenoma |
| 5 | Male | 46 | √ |  | √ | √ |  |  |  |
| 6 | Male | 63 |  |  | √ |  |  |  |  |
| 7 | Female | 66 | √ | √ | √ | √ |  |  |  |
| 8 | Male | 55 |  | √ | √ | √ | √ | Nonlateralized |  |
| 9 | Female | 38 | √ | √ | √ | √ | √ | Lateralized | Adrenal cortical adenoma |
| 10 | Female | 53 | √ |  | √ | √ |  |  |  |
| 11 | Female | 36 | √ | √ | √ |  | √ | Nonlateralized |  |
| 12 | Male | 38 | √ |  | √ |  |  |  |  |
| 13 | Male | 52 | √ |  | √ | √ |  |  |  |
| 14 | Male | 49 | √ | √ | √ | √ |  |  |  |
| 15 | Male | 38 | √ |  | √ |  |  |  |  |
| 16 | Female | 55 | √ | √ |  | √ |  |  |  |
| 17 | Male | 53 | √ |  | √ |  |  |  |  |
| 18 | Male | 58 | √ | √ | √ |  | √ | Nonlateralized |  |
| 19 | Male | 39 | √ |  | √ | √ |  |  |  |
| 20 | Male | 63 | √ | √ |  | √ |  |  |  |
| 21 | Female | 62 | √ | √ |  | √ |  |  |  |
| 22 | Female | 52 | √ | √ | √ |  |  |  |  |
| 23 | Female | 54 | √ | √ |  | √ |  |  |  |
| 24 | Female | 43 | √ |  | √ |  | √ | Nonlateralized |  |
| 25 | Male | 55 |  | √ | √ | √ | √ | Lateralized |  |
